# Why isn’t each cell its own cell type? Diminishing returns of increasing cell type diversity can explain cell type allometry

**DOI:** 10.3389/fcell.2022.971721

**Published:** 2022-10-10

**Authors:** Stefan Semrau

**Affiliations:** Leiden Institute of Physics, Leiden University, Leiden, Netherlands

**Keywords:** single-cell omics, cell type, allometry, power law, evolutionary fitness

## Abstract

Since the discovery of cells by Robert Hooke and Antoni van Leeuwenhoek in the 17th century, thousands of different cell types have been identified, most recently by sequencing-based single-cell profiling techniques. Yet, for many organisms we still do not know, how many different cell types they are precisely composed of. A recent survey of experimental data, using mostly morphology as a proxy for cell type, revealed allometric scaling of cell type diversity with organism size. Here, I argue from an evolutionary fitness perspective and suggest that three simple assumptions can explain the observed scaling: Evolving a new cell type has, 1. a fitness cost that increases with organism size, 2. a fitness benefit that also increases with organism size but 3. diminishes exponentially with the number of existing cell types. I will show that these assumptions result in a quantitative model that fits the observed cell type numbers across organisms of all size and explains why we should not expect isometric scaling.

## Introduction

**FIGURE 1:**
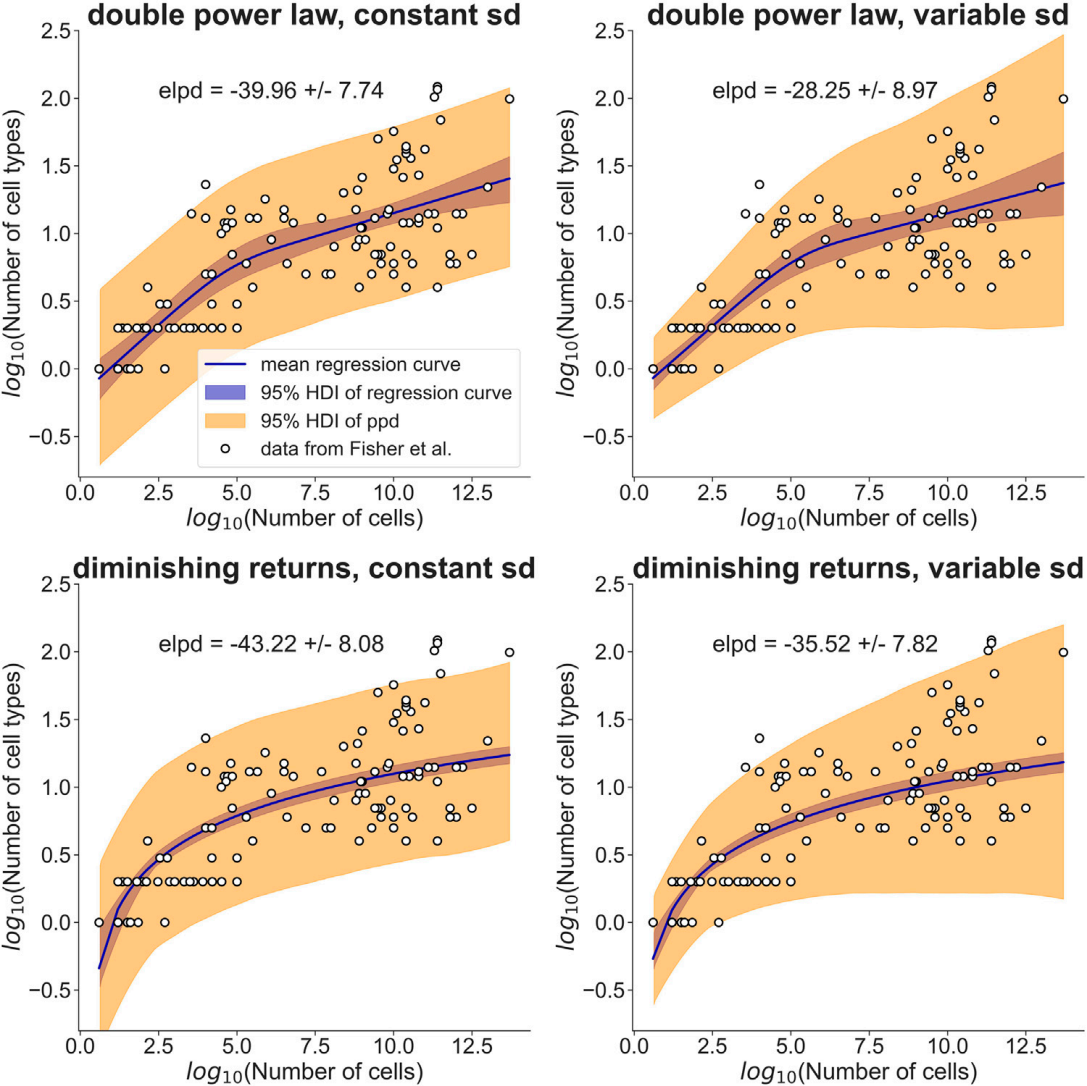
A simple model assuming diminishing fitness benefits of additional cell types performs as well as a double power law in explaining cell type allometry. Fisher et al. (2013, 2020)) collected cell type information for a range of different organisms (open circles). They modeled the observed allometric relationship with separate power laws for small and large organisms (top row). My model explains the observed allometry by a fitness benefit that diminishes with increasing cell type number (bottom row). I used a Bayesian hierarchical modeling approach to compare the two models. The data was assumed to be normally distributed with a standard deviation (sd) that was either constant (left column) or allowed to increase linearly with log_10_(Number of cells) (right column). The regression curves, shown as solid lines, are posterior means of the models, with the 95% highest density interval (HDI) indicated by a blue band. The orange band indicates the HDI of the posterior predictive distribution (ppd). For each model, the expected log predictive density (elpd) is reported together with its standard error. Judging by the elpd, the ‘diminishing returns’ model performs about as well as the double power law and the models with variable sd perform slightly better than the models with constant sd. Point estimates and HDIs of all parameters can be found in Table 1.

Since the advent of high throughput single-cell profiling techniques, a large number of cell types has been catalogued across many different tissues. For example, the Tabula Sapiens consortium recently identified over 400 cell types across 24 different human tissues (Tabula Sapiens Consortium et al., 2022). Whether each cluster of transcriptomes or other molecular profiles should be considered a separate cell type is still under debate (Clevers, 2017; Mircea and Semrau, 2021) and we certainly need improved methods to discriminate biologically meaningful variability from random noise (Mircea et al., 2022). Nevertheless, single-cell profiling has revealed a high diversity of cell states and one might be forgiven to wonder: Could each cell be its own, highly specialized cell type? Here, I will argue, from an evolutionary fitness perspective, that we should expect much fewer cell types than cells in an organism. Whole-organism single-cell transcriptomics data sets are currently still rare (Lähnemann et al., 2020) and, as mentioned above, uncertainties in the interpretation of these data sets remain. To circumvent these problems, I base my arguments on recent studies by Fisher et al. (Fisher et al., 2013; Fisher et al., 2020), who collected published cell type numbers, mostly derived from morphological characteristics. These studies found that the number of cell types scales allometrically with the total number of cells in the organism (Figure 1). Intriguingly, the data could not be fit by a single power law, in contrast to many other allometric relationships (West and Brown, 2005). As shown in seminal work by Geoffrey West and co-workers, power law scaling can arise from the optimization of metabolic rate subject to geometric constraints of relevant tissues, such as the vasculature (West et al., 1997; Enquist et al., 1999; West et al., 1999; West et al., 2002). Fisher et al. therefore fit two separate power laws, for small and large organisms, respectively, suggesting that larger organisms face additional constraints. In contrast to the allometric scaling of metabolic rate, it is not immediately obvious that geometric or physiological constraints should be the only relevant factors for cell type allometry. One might therefore not expect *a priori* to find power law scaling.

### Diminishing returns model

Here, I develop an alternative model that can explain the observed scaling across organisms of all sizes. This model considers the effect of a new cell type on an organism’s fitness. I adopt a notion of fitness described by Wagner as “a measure predicting the competitive ability of a genotype compared to another” (Wagner, 2010), which can in principle be determined by pairwise competition experiments. I reason that mutations giving rise to a new cell type can only be fixed in a population, if they lead to an increase in fitness. I therefore model the appearance of new cell types during evolution as discrete events that have an associated fitness cost ${\Delta f}_{\text{cost}}$ and benefit ${\Delta f}_{\text{benefit}}$ , which must result in a net-positive fitness change ${\Delta f=\Delta f}_{\text{benefit}}-{\Delta f}_{cost}$ . A common mechanism for the evolution of new cell types is the functional segregation of a multifunctional ancestor cell type into multiple sister cell types (Arendt, 2008). Such an event likely inflicts a, possibly small, fitness cost ${\Delta f}_{\text{cost}}$ . For example, if the number of cells remains constant, fewer cells will carry out each function of the multifunctional ancestor, which means that these functions might be impaired at the organismal level. Conversely, if the number of cells increases (to keep multiple functions at their original level), additional energy is needed. There might also be a small “overhead” related to the creation and organization of additional cell types during embryonic development as well as their ongoing regulation during an organism’s adult life. In these scenarios, a new cell type likely modulates an organism’s metabolic rate, which is known to obey power law scaling with cell number (West et al., 1997; Enquist et al., 1999; West et al., 1999; West et al., 2002). More generally, any fitness cost that is related to (bio)physical constraints likely scales in a similar way (Kempes et al., 2019). Hence, we model the fitness cost to scale like a power law with cell number N : ${\Delta f}_{\text{cost}}\propto N^{\delta }$ . For the same reasons, the fitness benefit ${\Delta f}_{\text{benefit}}$ provided by a functional segregation event is expected to obey a power law: ${\Delta f}_{\text{benefit}}\propto N^{\gamma }$ . Now, I posit that the fitness benefit should also depend on the number of already existing cell types. Given that more specialized cell types appear later in evolution and tend to provide functions that are refinements or variations of existing functions (Arendt, 2008), they likely confer a reduced fitness benefit compared to their predecessors. ${\Delta f}_{\text{benefit}}$ should therefore decline with the number of cell types *K*. If the fitness benefit declined as a power law (*K* ^−α^, α > 0), a single power law for *K* with respect to *N* would result. The fitness benefit must hence decline more quickly, i.e., exponentially. If the fitness benefit is reduced by a factor *b >* 1 at each segregation event, ${\Delta f}_{\text{benefit}}\propto N^{\gamma }b^{-K}$ . Taken together,

$${\Delta f=\Delta f}_{\text{benefit}}-{\Delta f}_{cost}=aN^{\gamma }b^{-K}-cN^{\delta }$$

Requiring Δf≿0 for a new cell type to appear leads to

$$b^{K}=\frac{a}{c}N^{\gamma -\delta }\Rightarrow K=\frac{\log_{10}\left(a/c\right)}{\log_{10}b}+\frac{\gamma -\delta }{\log_{10}b}\log_{10}N$$

$$\Rightarrow K=A+B\cdot \log_{10}N$$

$$with\;A=\frac{\log_{10}\left(a/c\right)}{\log_{10}b}\;,\;B=\frac{\gamma -\delta }{\log_{10}b}$$

$$\Rightarrow \log_{10}K=\log_{10}\;\left(A+B\cdot \log_{10}N\right)$$

$$\Rightarrow k=\log_{10}\left(A+B∙n\right)$$

$with\;k=\log_{10}\;K\;,\;n=\log_{10}\;N$

To rigorously compare this ‘diminishing returns’ model with the double power law, I used a Bayesian hierarchical approach (see Materials and Methods for the model definitions and priors). I assumed that the cell type numbers are normally distributed in log-space with a mean given by the double power law (i.e., a piecewise linear relationship in log-space) or the relationship derived above. Initially, I assumed the standard deviation to be constant (Figure 1, left column). Posterior distributions of the parameters were obtained by Markov Chain Monte Carlo sampling. Estimates of the slopes and breakpoint in the double power law were very similar to those obtained by Fisher et al. (Fisher et al., 2020) with ordinary least squares fitting (see Table 1, first two columns). To compare the models quantitatively I estimated the expected log posterior density (elpd) using leave-one-out cross-validation. The elpd was slightly larger for the double power law model but the difference was well within the standard error of the elpd (see Figure 1 and Table 1). The ‘diminishing returns’ model hence fits the data as well as the double power law. As the spread of the cell type numbers around the regression curves seems to increase with cell number, I next tested models in which the standard deviation was allowed to increase linearly with log-cell number (Figure 1, right column). Judging by the elpd, allowing the standard deviation to vary improved model performance for both the double power law and the ‘diminishing returns’ model (Table 1). Again, the difference in elpd between the double power law and the ‘diminishing returns’ model was within the standard error. The increased spread for larger organisms is possibly related to differences between multicellular lineages and the environments in which they evolved, as pointed out in Fisher et al (2020).

**TABLE 1:** Estimates of model parameters and model comparison. The power law parameters reported by Fisher et al, (2013); Fisher et al., 2020) (first column) are ordinary least-squares estimates and the intervals are confidence intervals (CIs). For the Bayesian models described in this paper (last 4 columns) parameters are given as the mean of the posterior together with the 95% highest density interval (HDI). The Bayesian models assume a normal distribution of the data in log space with the regression curve as the mean. *Intercept*, *slope (small N)*, *breakpoint* and *slope (large N)* parameterize the regression curve of the double power law, whereas *A* and *B* parameterize the regression curve of the ‘diminishing returns’ model, *sd* is the standard deviation of the normal distribution in the models that keep the standard deviation constant. *Sd intercept* and *sd slope* parametrize a linear increase of the standard deviation with log-cell number in the models that allow the standard deviation to vary. The expected log predictive density (elpd) and its standard error (se) was calculated for the Bayesian models using leave-one-out cross-validation.

	Fisher et al.	Double power law, constant sd	Diminishing returns, constant sd	Double power law, variable sd	Diminishing returns, variable sd
intercept k_0_ [HDI]		−0.20 [−0.41,−0.01]		−0.19 [−0.30,−0.08]	
slope (small N) s_small_ [CI or HDI]	0.21 [0.16,0.26]	0.21 [0.14,0.29]		0.20 [0.16,0.25]	
breakpoint n_bp_ [CI or HDI]	4.80 [3.90 5.70]	4.82 [2.81,6.64]		5.18 [3.52,6.84]	
slope (large N) s_large_ [CI or HDI]	0.07 [0.03 0.11]	0.07 [0.03,0.10]		0.06 [0.01,0.10]	
A [HDI]			−0.29 [−0.64,0.08]		−0.13 [−0.42,0.16]
B [HDI]			1.29 [1.09,1.49]		1.13 [0.94,1.33]
sd Σ [HDI]		0.32 [0.29 0.36]	0.33 [0.29,0.37]		
sd intercept Σ_0_ [HDI]				0.12 [0.06,0.19]	0.17 [0.10,0.24]
sd slope s_Σ_ [HDI]				0.03 [0.02,0.04]	0.02 [0.01,0.04]
elpd [se]		−39.96 [7.74]	−43.22 [8.08]	−28.25 [8.97]	−35.52 [7.82]

### Discussion

In the derivation presented here, I made several assumptions that require critical assessment. First, I implied that cell types are discrete and stable entities, while others put forward the notion of dynamic cell states that lie on a continuum (Clevers, 2017). I further assumed that cell types are functionally different, by some measure, and able to confer a fitness advantage when they appear. I treated cell morphology as a reasonable proxy for cell type, which might lead to an underestimation of the number of cell types. Likely, the number of observed morphologies is some fraction of the true number of cell types, such that the true scaling behavior is still qualitatively the same as observed by Fisher et al. In my model, the appearance of a new cell type is a discrete event, which is certainly a strong simplification of the actual processes by which new cell types arise (Arendt, 2008). Finally, I modeled the diminishing benefits provided by additional cell types with an exponential decay. While it is reassuring that the resulting model fits the data set considered here, direct fitness measurements will be necessary to confirm this assumption.

In summary, I developed a phenomenological model of cell type allometry using a minimal number of assumptions. The model is therefore agnostic of evolutionary lineages and related systematic differences. Nevertheless, I showed that diminishing fitness benefits can explain the observed cell type allometry. I hope that this manuscript will stimulate experiments and the development of more sophisticated models.

## Materials and methods

The experimental data shown in Figure 1 was published previously (Fisher et al., 2013) and made publicly available on Dryad (https://datadryad.org/stash/dataset/doi:10.5061/dryad.27q59). All models were fit in double log-space. Consequently, log-transformed cell numbers N and cell type numbers K are used in the model definitions:

$$\begin{array}{l} n=\log_{10\;}N\\ k=\log_{10\;}K \end{array}$$

To compare the double power law model with the ‘diminishing returns’ model, a Bayesian hierarchical approach was used. The log-cell type number *k* was assumed to be normally distributed. For the double power law, the mean of the normal distribution is given by a piecewise linear relationship between *n* and *k*. In the case of constant standard deviation (i.e., the spread of *k* does not depend on the log-cell number *n*), the double power law model is thus defined by

k∼Normal(μ=f(n),σ=Σ)

$$f\left(n\right)=k_{0}+\left\{ \begin{array}{l} s_{small}\cdot n\\ s_{small}\cdot n_{bp}+s_{large}\cdot \left(n-n_{bp}\right) \end{array}\right.\begin{array}{l} \mathrm{for}\;n<n_{bp}\\ \mathrm{for}\;n\geq n_{bp} \end{array}$$

$$k_{0}∼Uniform\left(a=-0.5,b=0.5\right)$$

$$s_{small}∼Normal\left(\mu =0,\sigma =20\right)$$

$$n_{bp}∼Normal\left(\mu =5,\sigma =2\right)$$

$$s_{large}∼Normal\left(\mu =0,\sigma =20\right)$$

Σ∼Halfcauchy(γ=10)

where *k*_0_ is the intercept of log-cell type numbers *k*, and s_small_ and s_large_ are the slopes below and above the breakpoint *n*_*bp*_, respectively. *Normal* indicates a normal distribution with mean μ and standard deviation σ, *Uniform* is a uniform distribution between *a* and *b*, and *HalfCauchy* is a Cauchy distribution at location 0 with half-width half-maximum γ that was truncated below 0 so that only positive values have non-zero probability.

For variable standard deviation (i.e., the spread of the log-cell type number *k* increases linearly with log-cell number *n*) the model is defined by

k∼Normal(μ=f(n),σ=g(n))

$$f\left(n\right)=k_{0}+\left\{ \begin{array}{cc} s_{small}\cdot n & for\;n<n_{bp}\\ s_{small}\cdot n_{bp}+s_{large}\cdot \left(n-n_{bp}\right) & for\;n\geq n_{bp} \end{array}\right.$$

$$k_{0}∼Uniform\left(a=0.5,b=0.5\right)$$

$$s_{small}∼Normal\left(\mu =0,\sigma =20\right)$$

$$n_{bp}∼Normal\left(\mu =5,\sigma =2\right)$$

$$s_{large}∼Normal\left(\mu =0,\sigma =20\right)$$

$$g\left(n\right)=\Sigma_{0}+s_{\Sigma }\cdot n$$

$$\Sigma_{0}∼Halfnormal\left(\sigma =1\right)$$

$$s_{\Sigma }∼Halfnormal\left(\sigma =1\right)$$

where Σ_0_ and s_Σ_ are the intercept and slope, respectively, of the linear model for the standard deviation. *HalfNormal* is a Normal distribution with mean μ = 0 and standard-deviation σ truncated below 0 such that only positive values have non-zero probabilities.

The ‘diminishing returns’ model, which assumes the fitness benefit to decrease with cell type number, is correspondingly defined by

k∼Normal(μ=f(n),σ=Σ)

$$f\left(n\right)=\log_{10\;}\left(A+B\cdot n\right)$$

A∼Normal(μ=0,σ=20)

B∼Normal(μ=0,σ=20)

Σ∼Halfcauchy(γ=10)

in the case of constant standard deviation and by

k∼Normal(μ=f(n),σ=g(n))

$$f\left(n\right)=\log_{10\;}\left(A+B\cdot n\right)$$

A∼Normal(μ=0,σ=20)

B∼Normal(μ=0,σ=20)

$$g\left(n\right)=\Sigma_{0}+s_{\Sigma }\cdot n$$

$$\Sigma_{0}∼Halfnormal\left(\sigma =1\right)$$

$$s_{\Sigma }∼Halfnormal\left(\sigma =1\right)$$

when the standard deviation is allowed to increase linearly with log-cell number *n*.

The posterior distributions of all parameters were obtained by Markov Chain Monte Carlo sampling using the python package *pymc* (version 4.1.2) with 2 chains, 2000 tuning steps and 10,000 samples. The “target_accept” parameter was kept at the default value of 0.8 except for the ‘diminishing returns’ model with constant standard deviation. That model required a “target_accept” of 0.99 to avoid divergences. For model comparison, the *arviz* python package (version 0.12.1) was used to estimate the expected log posterior density (elpd) by leave-one-out cross-validation. The regression curves shown as solid lines in Figure 1 are posterior means of *f(n)*: For each *n*, the average of *f(n)* over the posterior distribution of the parameters was calculated. The 95% highest density intervals (HDIs) shown as blue bands in Figure 1 correspond to the smallest intervals that contain 95% of the posterior distribution of *f(n)* for a specific *n*. The 95% HDIs of the posterior predictive distribution (ppd) correspond to the smallest intervals containing 95% of the posterior distribution of the log-cell type number *k* for a given *n*.

The jupyter notebook used to produce all presented results from the raw data can be obtained from github (https://github.com/semraulab/allometry).

## Data Availability

The dataset used in this study is publicly available from Dryad under a CC0 Universal (CC0 1.0) Public Domain Dedication license: https://datadryad.org/stash/dataset/doi:10.5061/dryad.27q59.
